# Comparison of good review practices of seven countries participating in the ECOWAS medicines regulatory harmonisation initiative: identifying opportunities for improvement

**DOI:** 10.3389/fmed.2024.1520892

**Published:** 2025-01-10

**Authors:** Mercy Owusu-Asante, Delese Mimi Darko, Seth Seaneke, Aminata Nacoulma, Oula Ibrahim Olivier Traore, Christianah Mojisola Adeyeye, Abayomi Akinyemi, Coulibaly Assane, Clarisse Épse Kaul Meledje Clamoungou, Oumy Kalsoum Ndao, Rokhaya Ndiaye Kande, James Komeh, Sheku Mansaray, Dalkoi Lamboni, Maheza Agba, Stuart Walker, Sam Salek

**Affiliations:** ^1^School of Life and Medical Sciences, University of Hertfordshire, Hatfield, United Kingdom; ^2^Food and Drugs Authority, Accra, Ghana; ^3^National Pharmaceutical Regulatory Agency, Ouagadougou, Burkina Faso; ^4^National Agency for Food and Drug Administration and Control, Abuja, Nigeria; ^5^Ministry of Public Health, Abidjan, Côte d'Ivoire; ^6^Ministry of Health and Social Welfare, Dakar, Senegal; ^7^Pharmacy Board of Sierra Leone, Freetown, Sierra Leone; ^8^Medicine and Laboratories, Lome, Togo; ^9^Centre for Innovation in Regulatory Science, London, United Kingdom; ^10^Institute of Medicines Development, London, United Kingdom

**Keywords:** Economic Community of West African States Medicines Regulatory Harmonisation (ECOWAS-MRH), good review practices, African Medicines Agency (AMA), regulatory reliance, Optimising Efficiencies in Regulatory Agencies (OpERA)

## Abstract

**Introduction:**

When implemented by national and regional regulatory agencies good review practices (GRevPs) support the timely high-quality review of medicines for enhanced patients’ availability to safe, quality and efficacious innovative and generic products. It is important that all aspects of GRevPs are continuously evaluated and updated to promote the continuous improvement of regulatory systems at national and regional levels. The aim of this study was to assess and compare the GRevPs of the national medicines regulatory agencies (NMRAs) of Burkina Faso, Cote d’Ivoire, Ghana, Nigeria, Senegal, Sierra Leone and Togo, who are active participants of the ECOWASMRH initiative to identify opportunities for improvement.

**Methods:**

The Optimising Efficiencies in Regulatory Agencies questionnaire, was completed by each of the NMRAs, which facilitates the assessment of GRevPs, which in turn affect the regulatory review processes.

**Results:**

Except for Cote d’Ivoire and Nigeria which are autonomous, the other five NMRAs operate within the administrative structure of their respective Health Ministry, to regulate medical products for human use, medical devices and diagnostics. Apart from Togo, the agencies receive partial funding from their governments as well as from regulatory fees. Population in the seven countries ranges from 8.6 million to 211.4 million. All the NMRAs had measures in place to achieve quality in their review processes, although there were some remaining initiatives related to transparency and communication, continuous improvement and training and education, to be implemented. Of the ten quality decision-making practices Ghana had implemented nine into a framework, Togo eight, Cote d’Ivoire seven, Nigeria six, and Burkina Faso five; while Sierra Leone has partially implemented all ten and Senegal had not implemented any of the quality decision-making practices.

**Conclusion:**

The study compared the organisation, GRevPs and quality decision-making processes of the NMRAs that actively participate in the ECOWAS-MRH initiative. Though some differences were identified with regard to organisation, a significant number of good review practice initiatives and quality decision-making practices were identified yet to be implemented to promote continuous improvement in the regulatory processes of the NMRAs.

## 1 Introduction

In 2015, the World Health Organization (WHO) issued guidelines on good review practices (GRevPs) for national and regional regulatory authorities for medical products to support the continual improvement of their effectiveness, efficiency and consistency. The review of medicines has been broadly defined by the WHO as “that part of the regulatory work that forms the scientific foundation for regulatory decisions on marketing authorizations. It requires a highly complex, multidisciplinary assessment of product data to ensure that products submitted for regulatory approval meet adequate scientific and evidentiary standards for safety, efficacy and quality” (1, 2).

GRevPs are defined by the WHO as “documented best practices for any aspect related to the process, format, content and management of a medical product review. The objective of GRevPs is to help achieve timeliness, predictability, consistency, transparency, clarity, efficiency and high quality in both the content and management of reviews. This is carried out through the development of guidelines, review tools (for example, standard operating procedures (SOPs) and templates) and reviewer learning activities (for example training courses, mentoring, orientation packages and discussion sessions). To promote continuous improvement, all aspects of GRevPs should be continuously evaluated and updated” (2). This definition has been supported and expanded by the European Medicines Agency, the United States Food and Drug Administration (3, 4).

The ten key principles of a good review are that it is balanced, considers context, is evidence-based, identifies signals, investigates and solves problems, makes linkages, utilizes critical analyses, is thorough, well-documented and well-managed activities, and guides regulatory authorities in their regulatory practices. Similarly, the benefits of implementing GRevPs by national and regional regulatory authorities include the timely quality review of medical products and the enhancement of patients’ availability to safe, quality and efficacious medicines in individual countries and regions (1).

Owing to the dynamic nature of the global regulatory landscape for medical products, it is necessary to assess the efficiencies of the relevant regulatory authorities available in the countries within the sub-region with a view to continually update the regulatory systems (3).

According to Al-Essa and colleagues, “quality measures may be evaluated on a regular basis to determine their impact on the quality and speed of the drug approval process. Review of human resources and the workload must always be assessed and updated according to the needs, challenges and opportunities for improving regulatory review practices” (3). Very useful insights on the implementation of quality measures by regulatory authorities have been provided by these same authors in their recent publication (3).

Therefore, in addition to assessing the quality measures, human resources and workload, this study will also assess transparency and communication parameters and continuous improvement initiatives, as well as training and education programmes.

To further highlight the regulatory importance of GRevPs, it was reported that the Asia Pacific Economic Cooperation (APEC) Regulatory Harmonization Steering Committee instituted the implementation of the 2020 Good Review Practices roadmap. Two international workshops were successfully organized by the Taiwan Food and Drug Administration including other objectives which addressed the building blocks of a regulatory review system in line with the roadmap. From the workshops it was noted that regulatory authorities associated the implementation of quality measures with efficient and transparent regulatory systems (5).

Lin and colleagues reported that “there is a lack of uniformity in review practices for medical products among APEC economies, as each economy has different regulatory practices, levels of expertise and capacity…” and “…the implementation of GRevP could be essential for strengthening the performance of regulatory authorities and enhancing mutual trust between economies in the APEC region” (5).

In the Economic Community of West African States-Medicines Regulatory Harmonisation (ECOWAS-MRH) initiative, there are seven national medicines regulatory agencies (NMRAs) that are active in the assessment of applications for marketing authorisation in the subregion. As all the 15 NMRAs in the ECOWAS region collaborate to implement this initiative, it is expected that assessing and improving the GRevPs in the seven active NMRAs will in turn benefit all the NMRAs in the ECOWAS region (6).

According to the WHO, “good communication is critical and has many advantages for regulatory authorities, applicants and the public. It can improve the efficiency of the development and review processes and thus ultimately speed up patients’ access to quality medical products” (1).

Because successful assessments of GRevPs of countries participating in the ZaZiBoNa and East African Community (EAC)-MRH initiatives have been conducted (7, 8) it is appropriate that the GRevPs of countries participating in the ECOWAS-MRH initiative are assessed. This study, therefore, is aimed at assessing those GRevPs and to communicate the findings to other regulatory authorities, stakeholders and the public to serve as a reference for future comparative analyses and to promote best practices in ECOWAS

This publication, which is one of a two-part series, provides an insight into the implementation of GRevPs of countries participating in the ECOWAS-MRH initiative. The other publication will compare their review models and regulatory timelines.

## 2 Methods and materials

### 2.1 Study participants

All seven active NMRAs of the ECOWAS-MRH initiative namely, National Pharmaceutical Regulatory Agency-Burkina Faso, Ministry of Public Health-Republic of Cote d’Ivoire, Food and Drugs Authority (Ghana-FDA), National Agency for Food and Drug Administration and Control (NAFDAC), The Federal Republic of Nigeria, Ministry of Health and Social Welfare, Republic of Senegal, Pharmacy Board of Sierra Leone (PBSL) and the Directorate of Pharmacy, Medicine and Laboratories-Togo, participated in this study between August 2021 and November 2023.

### 2.2 Data collection

The Optimising Efficiencies in Regulatory Agencies (OpERA) questionnaire was used to collect data. The development and validation of the OpERA Questionnaire followed the standard methodology for design of such tools. Initially, the content was based on a focus group of regulatory and pharmaceutical industry experts and then tested for validity and reliability in the field with the regulatory authorities and pharmaceutical companies as study participants. Completion of the OpERA questionnaire facilitates the assessment of the regulatory review processes, which affect approval times. Upon completion of the OpERA questionnaire, a country report, specific to each NMRA, is generated, which enables the sharing and adoption of GRevPs (9).

The OpERA questionnaire consists of six modules: module 1 covers structure, organisation and resources of the agency; module 2 explores the review models used for the scientific assessment of medicines; module 3 identifies the key milestones in the review process; module 4 captures regulatory measures that have been built into the regulatory review process; module 5 explores the quality of decision-making processes and module 6 documents the agency’s perception of the key drivers and barriers that influence the effectiveness and efficiency of its review and decision-making processes.

*While this manuscript covers the first three modules of the OpERA Questionnaire, because of the extensive nature of the remaining three modules including models of review, timelines (metrics) and challenges, it was agreed that these will be provided in a separate manuscript.*

## 3 Results

For the purpose of clarity, the results of this study cover three out of the six OpERA modules. These are presented in the following three parts: (1) Organisation of the authorities, (2) GRevPs building quality into the review process and (3) Quality decision-making processes.

### 3.1 Part 1. Organisation of the authorities

The NMRAs of Burkina Faso, Cote d’Ivoire, Ghana, Nigeria, Senegal, Sierra Leone and Togo were all established within a span of three decades (from 1992 to 2022). With the exception of Cote d’Ivoire and Nigeria, which are autonomous, the other NMRAs operate within the administrative structure of their respective Health Ministries. All the authorities regulate medical products for human use, medical devices and diagnostics. The population in the seven countries varies from 8.6 million to 211.4 million. A summary of the human resources of the NMRAs is provided in Table 1. The ratio of the staff per million residents ranged from 2.5 to 23.3, with five of the authorities having a ratio of less than 10. All the authorities, with the exception of Togo, receive partial funding from their governments as well as from regulatory fees. Table 2 details the fees charged for the review of marketing authorization applications for new active substances (NASs) and generics, respectively.

**Table 1:** Comparison of country population, NMRA size and workload in 2022.

Country	Burkina Faso	Cote d’Ivoire	Ghana	Nigeria	Senegal	Sierra Leone	Togo
Population (millions)	22.7	28.2	30.8	211.4	17.3	8.6	8.8
Number of staff	64	71	683	2080	50+	200	30
Staff per million residents	2.8	2.5	22.2	9.8	2.9	23.3	3.4
Number of internal reviewers	34	15	26	44	37	15	4
Reviewers in agency, %	53	21	3.8	2.1	74	7.5	13.3

**Table 2:** Comparison of fees charged and source of funding in 2022.

Country	Burkina Faso	Cote d’Ivoire	Ghana	Nigeria	Senegal	Sierra Leone	Togo
Source of funding	93% government, 7% fees	63% government, 37% fees	35% government, 65% fees	22.41% government, 77.59% fees, 5.5% international partners	government and fees	90% government, 10% donor funds	100% government
Fees for review of new active substances (USD)	494	808	1,080	1,280	2,511	750	327
Fees for review of generics (USD)	247	808	720	1,280	1,674	250	818

### 3.2 Part 2. GRevPs building quality into the review process

For the purpose of clarity, the documentation of review procedures that include general measures used to achieve quality, transparency and communication parameters, continuous improvement initiatives as well as training and education strategies that the authorities have in place, are presented as follows.

#### 3.2.1 General measures used to achieve quality

A summary of the comparison of the quality measures implemented by the NMRAs within the ECOWAS region is provided in Table 3.

**Table 3:** Comparison of the quality measures implemented by the NMRAs.

Indicator	NMRA						
	Burkina Faso	Cote d’ivoire	Ghana	Nigeria	Senegal	Sierra Leone	Togo
Good review practice system	✓	✓	✓	✓	✓	✓	✓
Internal quality policy	✓	✓	✓	✓	✓	✓	✓
Standard operating procedures (SOPs) for guidance of assessors	✓	✓	✓	✓	✓	✓	✓
SOPs for the advisory /registration committee consulted during the review process	✓	✓	✓	✓	✓	✓	x
Assessment templates	✓	✓	✓	✓	✓	✓	✓
Assessment report	✓	✓	✓	✓	✓	✓	✓
SOPs for completing the assessment report	✓	✓	✓	✓	✓	✓^a^	✓^a^
SOPs for any other procedures in the regulatory review process (e.g., validation)	✓	✓	✓	✓	✓	✓	✓^a^
Dedicated quality department	✓	✓	✓	✓	✓	✓	✓^a^
Scientific Committee	✓	✓	✓	✓	✓	✓	✓
Shared and joint reviews	✓	✓	✓	✓	✓	✓	✓

^aImplemented but not formally documented.^

All the authorities have measures in place to achieve quality in their review processes namely; a good review practice system, an internal quality policy, standard operating procedures (SOPs) for the guidance of assessors, SOPs for the advisory and /or registration committee consulted during the review process, assessment templates, assessment report, SOPs for completing the assessment report, SOPs for any other procedures in the regulatory review process, a dedicated quality department, a scientific committee and also shared and joint reviews. Only Togo has a few of the quality measures that are informally implemented; however, SOPs for the advisory committee are not in place.

#### 3.2.2 Transparency and communications parameters

A summary of the comparison of the transparency and communication parameters implemented by the NMRAs within the ECOWAS initiative is provided in Table 4.

**Table 4:** Comparison of the transparency and communication parameters implemented by the NMRAs.

Indicator	NMRA						
	Burkina Faso	Cote d’ivoire	Ghana	Nigeria	Senegal	Sierra Leone	Togo
Post-approval feedback to applicant on quality of submitted dossiers	✓	✓	✓	x	✓	✓	✓
Details of technical staff to contact	x	x	✓^a^	x	✓	✓^a^	✓^a^
Pre-submission scientific advice to industry	x	x	✓^a^	✓	x	✓	✓
Official guidelines to assist industry	x	✓	✓	✓	x	✓	✓
Industry can track progress of applications	✓	✓^a^	✓	✓	✓	✓^a^	✓^a^
Publication of summary grounds on which approval was granted	✓	✓	✓	x	x	✓	✓
Approval times	✓	✓	✓	✓	x	✓	✓
Advisory committee meeting dates	✓	✓	✓	x	x	✓	✓
Approval of products	✓	✓	✓	✓	✓	✓	✓^a^

^aImplemented but not formally documented.^

It was noted that out of the nine listed parameters, Ghana and Sierra Leone have formally implemented seven and informally implemented the remaining two parameters. Burkina Faso, Cote d’Ivoire and Togo have also implemented six parameters. Nigeria and Senegal have formally implemented five and four parameters, respectively.

#### 3.2.3 Continuous improvement initiatives

Sierra Leone is the only country that has formally implemented all the five listed parameters in line with continuous improvement initiatives. Nigeria and Senegal have formally implemented four of the parameters and Cote d’Ivoire and Togo have informally implemented one and two parameters, respectively. A summary of the comparison of the continuous improvement initiatives implemented by the NMRAs is provided in Table 5.

**Table 5:** Comparison of the continuous improvement initiatives implemented by the NMRAs.

Indicator	NMRA						
	Burkina Faso	Cote d’ivoire	Ghana	Nigeria	Senegal	Sierra Leone	Togo
External peer review	x	x	x	x	x	✓	x
Internal peer review	x	✓^a^	✓	✓	✓	✓	x
Internal tracking systems	✓^a^	x	x	✓	✓	✓	✓^a^
Review of assessors’ feedback	✓	x	✓	✓	✓	✓	x
Review of stakeholders’ feedback	✓	x	✓	✓	✓	✓	✓^a^

^aImplemented but not formally documented.^

#### 3.2.4 Training and education strategies

A summary of the comparison of the training and education strategy implemented by the NMRAs is provided in Table 6. It was noted that Ghana and Sierra Leone have formally implemented all the nine listed initiatives. Senegal has formally implemented seven of the initiatives while Cote d’Ivoire has informally implemented seven of the initiatives. Burkina Faso and Togo have only implemented three initiatives.

**Table 6:** Comparison of the training and education strategies implemented by the NMRAs.

Indicator	NMRA						
	Burkina Faso	Cote d’ivoire	Ghana	Nigeria	Senegal	Sierra Leone	Togo
Training programme for assessors	x	✓^a^	✓	✓	✓	✓	x
International workshops/conferences	x	✓^a^	✓	✓	✓	✓	x
External courses	x	✓^a^	✓	x	✓	✓	x
In-house courses	x	✓^a^	✓	x	✓	✓	x
On-the-job training	✓^a^	✓^a^	✓	✓	✓	✓	x
External speakers invited to the authority	x	✓^a^	✓	x	x	✓	✓
Induction training	✓	✓^a^	✓	✓	✓	✓	✓^a^
Sponsorship of post-graduate degrees	✓^a^	x	✓	x	x	✓	✓
Placements and secondment in other regulatory agencies	x	x	✓	✓	✓	✓	x

^aImplemented but not formally documented.^

### 3.3 Part 3. Quality decision-making processes

According to the WHO guidelines, NMRAs are encouraged to have a framework in place that forms the basis of the quality decision-making practices (QDMPs) to approve or reject a marketing authorisation application (2). The following ten principles should be implemented into the framework and also adhered to in practice: namely have a systematic, structured approach, assign clear roles and responsibilities(decision makers, advisors, information providers), assign values and relative importance to decision criteria, evaluate both internal and external influences/biases, examine alternative solutions, consider uncertainty, re-evaluate as new information becomes available, perform impact analyses of the decision, ensure transparency and provide a record trail and finally effectively communicate the basis of the decision (10, 11).

It was noted from the study that Ghana has implemented nine of the ten quality decision-making practices into a framework and additionally these nine practices are also adhered to in practice. Togo and Cote d’Ivoire have implemented eight and seven of the quality decision-making practices into a framework, respectively. Nigeria and Burkina Faso have implemented six and five of the quality decision-making practices into a framework, respectively, and additionally these practices are also adhered to in practice.

Sierra Leone has partially implemented all ten quality decision-making practices into a framework and has also partially adhered to the practices. Senegal has neither implemented quality decision-making practices into a framework nor adhered to these quality decision-making practices. A summary of the comparison of the quality decision-making practices implemented by the NMRAs is provided in Table 7.

**Table 7:** Comparison of the quality decision-making practices implemented by the NMRAs.

Practice	Burkina Faso		Ghana		Nigeria		Cote d’Ivorie		Senegal		Sierra Leone		Togo	
	Implemented into framework	Adhered to in practice	Implemented into framework	Adhered to in practice	Implemented into framework	Adhered to in practice	Implemented into framework	Adhered to in practice	Implemented into framework	Adhered to in practice	Implemented into framework	Adhered to in practice	Implemented into framework	Adhered to in practice
Have a systematic structured approach	✓	✓	✓	✓	✓	✓	✓	✓	NV	NV	✓ (in progress)	✓ (in progress)	✓	✓
Assign clear roles and responsibilities	✓	✓	✓	✓	✓	✓	✓	✓	×	NV	✓ (in progress)	✓ (in progress)	✓	✓
Assign values and relative importance to decision criteria	✓	✓	✓	✓	✓	✓	✓	✓	NV	NV	✓ (in progress)	✓ (in progress)	✓	✓
Evaluate both internal and external influences/biases	✓	NV	✓	✓	✓ (in progress)	✓ (in progress)	×	×	NV	NV	✓ (in progress)	✓ (in progress)	✓	✓
Examine alternative solutions	✓	NV	✓	✓	✓ (in progress)	✓ (in progress)	✓ (in progress)	✓	✓	NV	✓ (in progress)	✓ (in progress)	×	×
Consider uncertainty	×	NV	✓	✓	✓ (in progress)	✓ (in progress)	✓	✓ (in progress)	NV	NV	✓ (in progress)	✓ (in progress)	✓	✓
Re-evaluate as new information becomes available	×	NV	✓	✓	✓	✓	✓	✓	NV	NV	✓ (in progress)	✓ (in progress)	✓	✓
Perform impact analysis of the decision	×	NV	✓ (in progress)	✓ (in progress)	✓ (in progress)	✓ (in progress)	×	×	NV	NV	✓ (in progress)	✓ (in progress)	×	×
Ensure transparency and provide a record trail	✓	✓	✓	✓	✓	✓	✓	✓	NV	NV	✓ (in progress)	✓ (in progress)	✓	✓
Effectively communicate the basis of the decision	✓	✓	✓	✓	✓	✓	✓	✓	NV	NV	✓ (in progress)	✓ (in progress)	✓	✓

## 4 Discussion

This study compared the GRevPs of countries participating in the ECOWAS-MRH initiative and identified opportunities for improvement. The analysis, which is similar to the Southern Africa Development Community (SADC) (8) and EAC (7) regional studies, was also designed to widely share the regulatory good practices in the ECOWAS region to all stakeholders. These practices could interest manufacturers in increasing investment in the region for the ultimate benefit to patients.

It is of interest to note that out of the seven NMRAs, Nigeria and Ghana had the lowest percentage of reviewers in their authorities. It was also noted that Nigeria and Ghana had the highest contribution of their funds from regulatory fees. Coincidentally, Nigeria and Ghana have achieved WHO Global Benchmarking Tool maturity level-3 status, signifying that they have stable, well-functioning and integrated regulatory systems. It can therefore be inferred that these two authorities are demonstrating efficiency in utilizing their human and financial resources to strengthen their regulatory systems. This could serve as a major learning point for other NMRAs who seek to make improvements to their regulatory systems.

The ratio of the staff per million residents in five of the authorities was less than 10, similar to that reported by Sithole and colleagues with regard to the SADC region (8); only two authorities had a staff per million residents’ ratio of about twenty.

The lack of autonomy for most NMRAs in the ECOWAS region is a major challenge that also exists in the EAC and SADC regions (7, 8) and relevant provisions have been made in the African Union Model Law to promote the autonomous NMRAs, enabling independent decision making as well as their financial structure.

This study assessed the regulatory GRevPs of these NMRAs with regard to the implementation of quality measures, transparency and communication parameters, continuous improvement initiatives and training and education programmes. It was noted that the quality measures had been largely implemented by the NMRAs within the ECOWAS region, serving as a useful reference for other NMRA implementation. Some transparency and communication parameters remain to be implemented by the ECOWAS-MRH authorities, presenting an opportunity for the exchange of strategies in order for each of the NMRAs to implement all remaining parameters. Analysis further revealed that Sierra Leone was the only studied country that has fully implemented all continuous improvement initiatives at this time, representing another instance for potential learning for other authorities in the region. According to O’Brien and associates, *“*Regulators may elect to use external experts from academia, external experts must have appropriate knowledge, skills and experience to conduct an assessment; have no conflicts of interest; meet pre-agreed deadlines and respect the confidentiality of data” (12). Finally, comparing the training and education initiatives that have been implemented by the NMRAs showed that implementation of these programmes in Sierra Leone and Ghana could both serve as references to the other authorities in the region. There appears to be a correlation between implementation of training and education initiatives with the number of staff. This study shows that due to the relatively small number of staff in these agencies, Sierra Leone and Ghana have prioritised the implementation of training and education initiatives to improve GRevPs in their respective authorities.

This study has therefore shown that resources are available in the ECOWAS region for the NMRAs to rely on to improve their respective GRevPs; however, since it was also demonstrated that none of the NMRAs had fully implemented a quality decision-making framework nor had fully adhered to these decision-making practices, this can be considered to be a challenge that needs to be resolved.

## 5 Recommendations

The following are the recommendations for improving the GRevPs of countries participating in the ECOWAS-MRH initiative.

- *Autonomy of regulatory authorities*: The NMRAs in the ECOWAS region should work towards achieving autonomy, enabling them to have independent decision-making as well as having appropriate financial structure.
- *Regulatory strengthening:* Consideration should be given to employing the services of external experts for the review of marketing authorisation applications in view of the limited resources currently within some of the NMRAs in the ECOWAS region.
- *Transparency and communication strategies:* Authorities in the region would benefit from implementing additional good review practice measures as well as sharing of assessment reports with applicants.
- *Quality decision-making practices:* It is recommended that all authorities implement the 10 quality decision-making practices underpinned by initiating appropriate structured training.

## 6 Conclusion

This comparative study of the GRevPs of countries participating in the ECOWAS-MRH initiative has highlighted both the similarities among the authorities and also the differences that should be addressed in order to improve the regulatory systems in these countries. The full implementation of GRevP should be essential for strengthening the performance of regulatory authorities and enhancing mutual trust between the NMRAs in the ECOWAS region.

## Data Availability

The raw data supporting the conclusions of this article will be made available by the authors, without undue reservation.
